# Feasibility and Safety of Permanent Left Bundle Branch Pacing in Patients With Conduction Disorders Following Prosthetic Cardiac Valves

**DOI:** 10.3389/fcvm.2021.705124

**Published:** 2021-08-17

**Authors:** Hui-Qiang Wei, Hui Li, Hongtao Liao, Yuanhong Liang, Xianzhang Zhan, Qianhuan Zhang, Hai Deng, Wei Wei, Zili Liao, Yang Liu, Fangzhou Liu, Weidong Lin, Yumei Xue, Shulin Wu, Xianhong Fang

**Affiliations:** Department of Cardiology, Guangdong Cardiovascular Institute, Guangdong Provincial People's Hospital, Guangdong Academy of Medical Sciences, Guangzhou, China

**Keywords:** left bundle branch pacing, prosthetic valves, physiological pacing, conduction system, pacing

## Abstract

**Background:** The feasibility and safety of left bundle branch pacing (LBBP) in patients with conduction diseases following prosthetic valves (PVs) have not been well described.

**Methods:** Permanent LBBP was attempted in patients with PVs. Procedural success and intracardiac electrical measurements were recorded at implant. Pacing threshold, complications, and echocardiographic data were assessed at implant and follow-up visit.

**Results:** Twenty-two consecutive patients with atrioventricular (AV) conduction disturbances (10 with AV nodal block and 12 with infranodal block) underwent LBBP. The PVs included aortic valve replacement (AVR) in six patients, mitral valve repair or replacement (MVR) with tricuspid valve ring (TVR) in four patients, AVR with TVR in one patient, AVR with MVR plus TVR in three patients, transcatheter aortic valve replacement (TAVR) in five patients, and MVR alone in three patients. LBBP succeeded in 20 of 22 (90.9%) patients. LBB potential was observed in 15 of 22 (68.2%) patients, including 10 of 15 (66.7%) patients with AVR/TAVR and five of seven (71.4%) patients without AVR/TAVR. AVR and TVR served as good anatomic landmarks for facilitating the LBBP. The final sites of LBBP were 17.9 ± 1.4 mm inferior to the AVR and 23.0 ± 3.2 mm distal and septal to the TVR. The paced QRS duration was 124.5 ± 13.8 ms, while the baseline QRS duration was 120.0 ± 32.5 ms (*P* = 0.346). Pacing threshold and R-wave amplitude at implant were 0.60 ± 0.16 V at 0.5 ms and 11.9 ± 5.5 mV and remained stable at the mean follow-up of 16.1 ± 10.8 months. No significant exacerbation of tricuspid valve regurgitation was observed compared to baseline.

**Conclusion:** Permanent LBBP could be feasibly and safely obtained in the majority of patients with PVs. The location of the PV might serve as a landmark for guiding the final site of the LBBP. Stable pacing parameters were observed during the follow-up.

## Introduction

Traditional right ventricular (RV) apical pacing has been widely used for about 50 years. However, long-term RV apical pacing is associated with increased risk for atrial fibrillation, heart failure, and mortality due to ventricular electrical and mechanical asynchrony (1, 2). Pacing at alternative RV sites, such as the septal or outflow tract pacing, has not been shown to be superior to RV apical pacing (3, 4). His bundle pacing (HBP) is a more physiologic form of pacing and has demonstrated a reduced risk of pacing-induced cardiomyopathy, heart failure hospitalization, and mortality compared with RV pacing (5–7). However, several factors, such as higher capture threshold, low R-wave amplitude, and longer learning curve, have limited the wider adoption of this technique in routine practice. Left bundle branch pacing (LBBP), a novel pacing strategy, has been considered to be a feasible and safe approach with low and stable pacing threshold and narrow QRS duration (8, 9).

Conduction system disease is not uncommon after prosthetic valve (PV) surgery. Recently, His–Purkinje conduction system pacing in patients with transcatheter aortic valve replacement (TAVR) has been reported (10). However, the feasibility and success rate of LBBP in patients with other PV surgery have not been well described. The aim of this study was to report the feasibility and safety of LBBP in patients undergoing pacemaker (PM) implantation for atrioventricular (AV) conduction diseases after PV surgery.

## Methods

### Patient Population

All patients who received an implantable PM after PV surgery for AV conduction diseases and underwent attempts at LBBP between January 2018 and December 2019 were retrospectively included in a single-center study. Patients were excluded from the study if they underwent pulse generator changes or cardiac resynchronization therapy (CRT) or implantable cardioverter defibrillator (ICD) implantation. This study was approved by the institutional review board.

### Preprocedural Management

All patients underwent a transthoracic echocardiography to access ventricular structure before the procedure. For patients under warfarin, uninterrupted administration was performed. All patients signed an informed consent prior to the procedure.

### Implantation Procedure

Intracardiac electrograms from the pacing lead and 12-lead ECG were continuously recorded in an electrophysiology recording system (LabSystem PRO, Bard Electrophysiology, Lowell, MA, USA).

LBBP was performed using the 3,830 pacing lead (SelectSecure, Medtronic, Minneapolis, MN, USA), which was delivered through a fixed-curve sheath (C315 His, Medtronic, Minneapolis, MN, USA) inserted *via* the left subclavian or axillary vein. The distal His location was identified at the right anterior oblique (RAO) 30°position, and the fluoroscopic view was used as a reference. Subsequently, the sheath with the lead was further advanced to the anterior lower site of the distal His position and rotated in a counterclockwise fashion to place the lead tip in a perpendicular orientation toward the interventricular septum (IVS). A “W” pattern by unipolar pacing in lead V_1_ was selected as the initial implantation site. As the lead tip was gradually screwed into the IVS, a rightward shift of the second notch in the “W” -shaped pacing morphology was recorded. Once the right bundle branch block (RBBB) pattern in lead V_1_ was observed, the rotation of the lead was stopped. The depth of the lead within the IVS was accessed by contrast injection *via* the sheath at the left anterior oblique (LAO) 45°fluoroscopic view. Left bundle branch (LBB) potential was usually recorded in patients without left bundle branch block (LBBB). Selective LBBP was defined as follows: (1) There was an isoelectric interval between pacing spike and ECG QRS complex; (2) The pacing spike–QRS interval was almost identical with the LBB potential–QRS interval; (3) A local ventricular intracardiac electrogram (EGM) was present as a discrete component. Nonselective LBBP was defined as the following criteria: (1) There was no isoelectric interval between pacing spike and ECG QRS complex; (2) The local ventricular EGM showed direct capture of local myocardium by the pacing stimulus.

### Data Collection and Follow-Up

**Figure 1:**
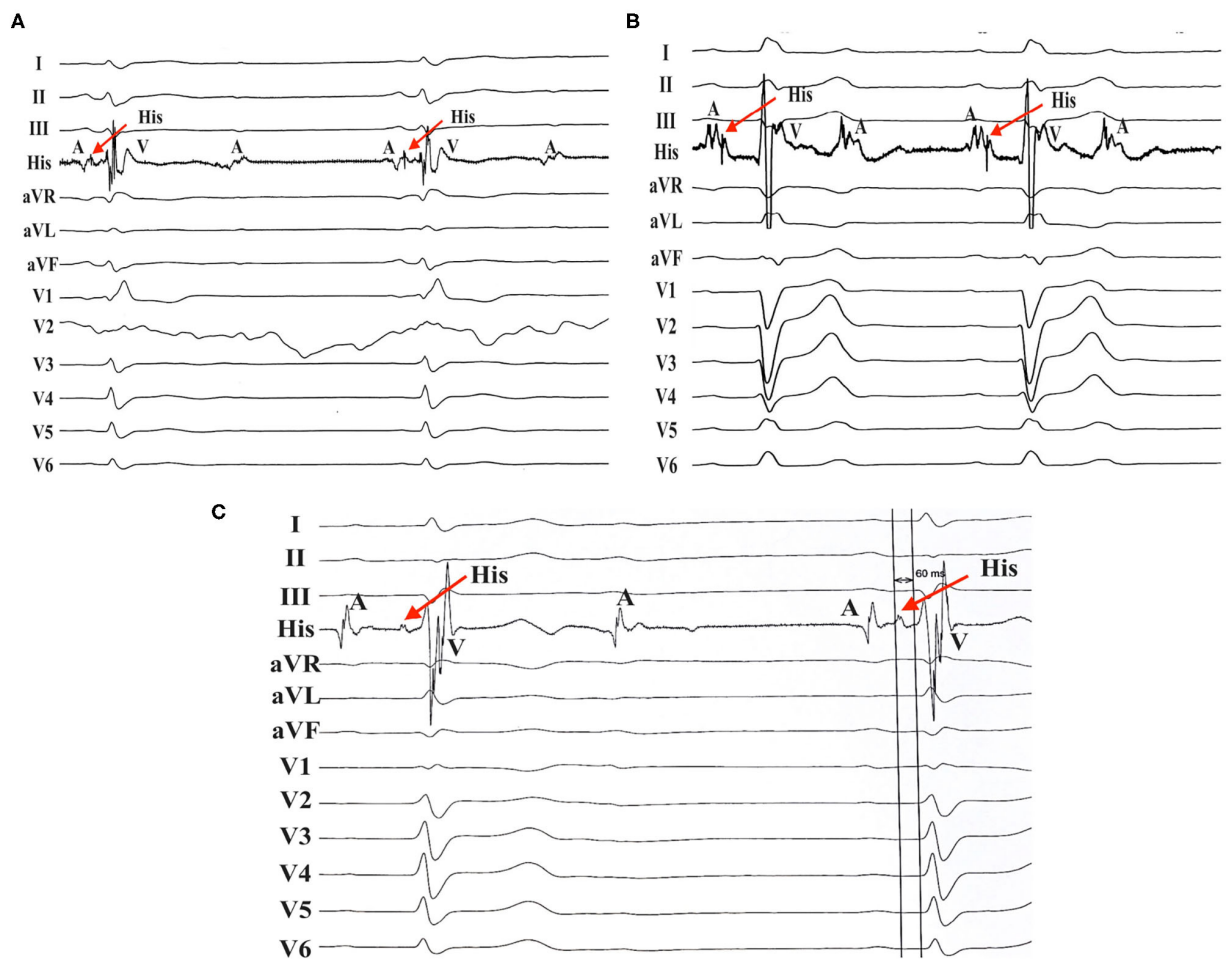
AV nodal block in patients with RBBB/LBBB. **(A)** Patient no. 8 with RBBB developed AV nodal block. **(B)** AV nodal block was found in patient no. 13 with LBBB. **(C)** Patient no. 17 with RBBB also developed AV nodal block with an His-ventricular (HV) interval of 60 ms. AV, atrioventricular; RBBB, right bundle branch block; LBBB, left bundle branch block.

Baseline characteristics and type of AV conduction diseases (AV nodal or infranodal) were documented. The type of AV conduction diseases was based on the intracardiac recording. The atrial deflection was not followed by a His potential, while the ventricular deflection was preceded by the His potential, which was defined as AV nodal block (Figure 1). The atrial deflection was followed by a conducted His potential, and ventricular deflection was not preceded by the His potential, which was characterized as infranodal block. Baseline echocardiographic parameters, such as the IVS thickness, left ventricular (LV) end-diastolic diameter, left ventricular ejection fraction (LVEF), and the degree of tricuspid regurgitation (TR) at baseline, were noted. Feasibility and LBBP parameters including the LBB capture threshold, R-wave amplitude, and pacing impedance were recorded. Presence of LBB potential, paced QRS duration, and the stimulus to peak left ventricular activation time (LVAT) were also recorded. The stim-LVAT was defined as the pacing stimulus to the peak of R-wave in lead V5 or V6. After discharge from the hospital, patients were scheduled for follow-up visit at 1, 3, 6, and 12 months in the device clinic. Pacing parameters were also recorded, and complications such as loss of capture, lead dislodgment, and significant increases in pacing threshold were tracked during the follow-up visit. Furthermore, multiple echocardiographic views were performed to quantify TR and to assess if there was any obstruction of tricuspid leaflet motion induced by the septal pacing lead. TR was categorized as none, mild, moderate, and severe. The severity of TR after LBBP lead implantation was compared to baseline echocardiography.

### Statistical Analysis

Continuous variables are given as mean ± standard deviation. The chi-square or Fisher exact test was used for categorical variables. Normally distributed continuous variables were analyzed using independent-sample *t*-test. Two-tailed paired *t*-test was performed for continuous paired variables. All statistical analyses were performed using SPSS 21.0 (SPSS, Inc., Chicago, IL, USA). A two-sided *p* < 0.05 was considered statistically significant.

## Results

### Baseline Characteristics

A total of 22 consecutive patients (14 males, 61.7 ± 13.5 years) who had undergone an attempt at LBBP were included during the study period. Four (18.2%) patients had hypertension, three (13.6%) patients had coronary disease, and six (27.3%) patients had atrial fibrillation. The mean LVEF at baseline was 61.0 ± 10.8%, with underlying LV dysfunction in 9.1% of patients. All patients had AV conduction diseases, with high-grade or complete AV nodal block in 10 patients and infranodal block in 12 patients. Baseline QRS duration was 120.0 ± 32.6 ms, with right bundle branch block (RBBB) in nine patients and left bundle branch block (LBBB) in five patients. Baseline characteristics and procedural results of each patient are described in Table 1.

**Table 1:** Baseline characteristics and procedural results.

**Patient**	**Age**	**Gender**	**Valve type**	**Site of block**	**AF**	**CMP**	**LVEF**	**Baseline QRSd (ms)**	**BBB**	**Paced QRSd (ms)**	**Septal thickness (mm)**
1	52	Female	AVR+MVR+TVR	AVN	Yes		62	92		116	8
2	54	Female	AVR	Infranodal			67	130	cRBBB	144	13
3	83	Male	TAVR	Infranodal			68	95		92	9
4	66	Female	AVR	Infranodal			70	84	iRBBB	106	9
5	49	Male	MVR	AVN			59	108		100	9
6	36	Male	AVR	Infranodal			75	156	cRBBB	128	10
7	72	Female	MVR+TVR	AVN			62	76		126	10
8	52	Male	MVR+TVR	AVN			45	164	cRBBB	120	11
9	40	Female	AVR+TVR	AVN			67	82	iRBBB	134	11
10	86	Female	AVR	AVN			70	88		130	12
11	55	Male	MVR	AVN			47	103		128	12
12	66	Female	MVR	AVN			69	98		132	10
13	62	Male	MVR+TVR	AVN	Yes	Yes	40	132	LBBB	133	8
14	61	Male	AVR	Infranodal			68	156	cRBBB	126	10
15	82	Male	TAVR	Infranodal			52	190	LBBB	146	12.7
16	61	Male	AVR+MVR+TVR	Infranodal	Yes		60	142	cRBBB	128	10
17	45	Male	MVR+TVR	AVN	Yes		55	138	cRBBB	142	11.7
18	57	Male	AVR	Infranodal			64	106		123	9
19	71	Male	TAVR	Infranodal			65	140	LBBB	128	13
20	61	Male	TAVR	Infranodal	Yes	Yes	38	148	LBBB	148	10
21	76	Female	TAVR	Infranodal			78	149	LBBB	138	10
22	70	Male	AVR+MVR+TVR	Infranodal	Yes		62	160	cRBBB	112	11

*AF, atrial fibrillation; AVN, atrioventricular nodal; AVR, aortic valve replacement; BBB, bundle branch block; CMP, cardiomyopathy; cRBBB, complete right bundle branch block; iRBBB, incomplete right bundle branch block; LBBB, left bundle branch block; LBBP, left bundle branch pacing; LVEF, left ventricular ejection fraction; MVR, mitral valve replacement or repair; TAVR, transcatheter aortic valve replacement; TVR tricuspid valve ring*.

### Implantation Results

The PVs included aortic valve replacement (AVR) in six patients, mitral valve repair or replacement (MVR) with tricuspid valve ring (TVR) in four patients, AVR with TVR in one patient, AVR with MVR plus TVR in three patients, TAVR in five patients, and MVR alone in three patients. Types of aortic valves implanted were the Sapien valve (Edwards Lifesciences, Irvine, CA, USA) in one patient, J-valve (Jiecheng Medical, Soochow, China) in one patient, and Venus-A valve (Venus Medtech, Hangzhou, China) in three patients. Twelve of 15 (80%) patients with AVR/TAVR developed infranodal block, while infranodal block was not found in patients without AVR/TAVR. Seventeen patients received a dual-chamber PM, whereas a single-chamber PM was implanted in five patients. LBBP was successfully obtained in 20 of 22 (90.9%) patients. In two patients (patients no. 2 and no. 15), we were unable to place the lead in the LV septum and the lead tip remained on the right side of the septum. AVR and TVR acted as good anatomic landmarks for guiding the LBBB lead implantation in our study. The final sites of LBBP were 17.9 ± 1.4 mm inferior to the AVR and 23.0 ± 3.2 mm distal and septal to the TVR. Contrast septal angiography was performed to confirm the depth of the lead in the septum in 15 of 22 (68.2%) patients in LAO fluoroscopic view. The mean length of the lead in the septum from the RV to LV wall along the course of the lead was 1.2 ± 0.36 cm.

### Electrophysiologic Characteristics

**Figure 2:**
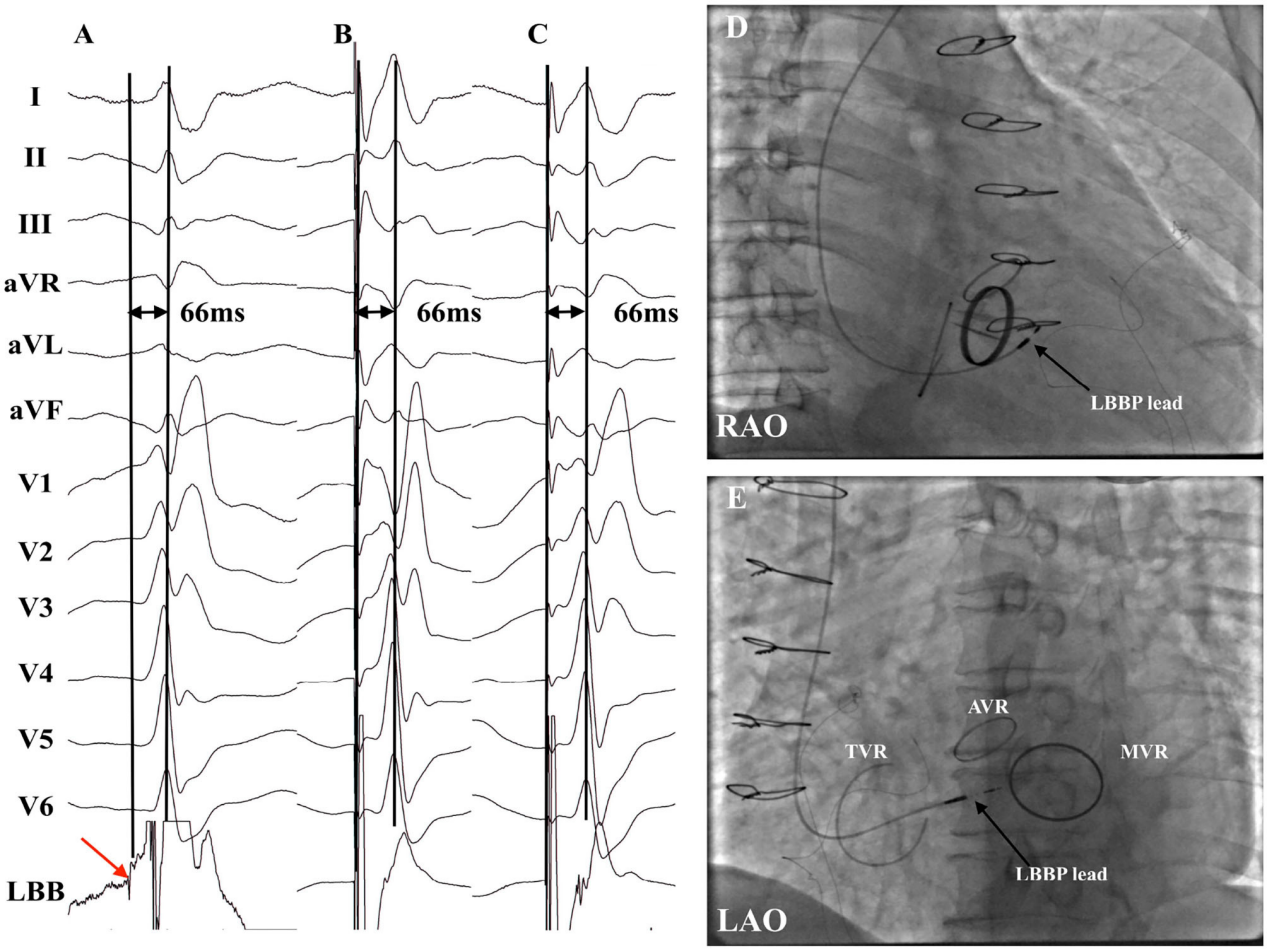
Successful LBBP in a patient with AVR with MVR plus TVR (patient no. 16). **(A)** LBB potential was recorded with the intrinsic LVAT being 66 ms. **(B)** Pacing at 3 V demonstrated nonselective LBBP with the stim-LVAT of 66 ms. **(C)** Selective LBBP was achieved under the low output at 0.5 V with the identical stim-LVAT of 66 ms. **(D,E)** Fluoroscopic images of LBBP in RAO view **(D)** and LAO view **(E)** revealed that the LBBP lead tip was distal and septal to the TVR and inferior to the AVR. AVR, aortic valve replacement; LAO, left anterior oblique; LBB, left bundle branch; LBBP, left bundle branch pacing; LVAT, left ventricular activation time; MVR, mitral valve replacement or repair; PV, prosthetic valve; RAO, right anterior oblique; TVR, tricuspid valve ring.

**Figure 3:**
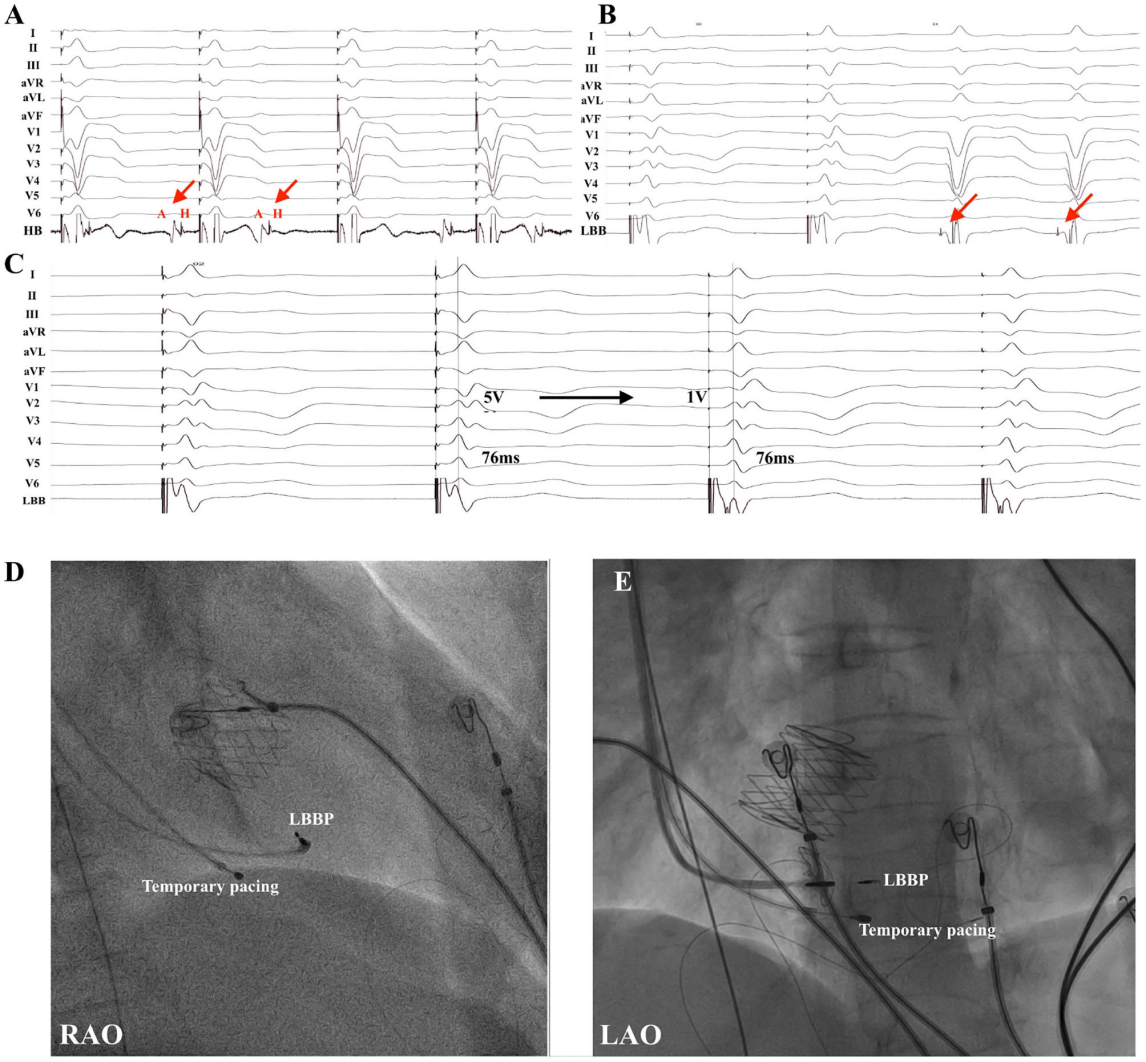
LBBP in a patient with TAVR (patient no. 19). **(A)** Example of a 71-year-old male who underwent TAVR and developed infranodal block. **(B)** Intrinsic rhythm with LBBB occurred after cessation of temporary right ventricular pacing and LBB potential (red arrow) was noted. **(C)** Pacing at 5 V resulted in correction of LBBB with nonselective LBBP with local myocardial fusion and stim-LVAT of 76 ms. Selective LBBP with discrete local ventricular myocardial electrogram was shown during pacing at 1 V with the same stim-LVAT of 76 ms. Note the subtle change in QRS morphology in lead V1 and intracardiac electrograms. **(D,E)** Fluoroscopic views of LBBP in a patient with TAVR were presented in RAO **(D)** and LAO **(E)** projections. HB, His bundle; LAO, left anterior oblique; LBB, left bundle branch; LBBB, left bundle branch block; LBBP, left bundle branch pacing; LVAT, left ventricular activation time; RAO, right anterior oblique; TAVR, transcatheter aortic valve replacement.

LBB potential was observed in 15 of 22 (68.2%) patients. Of these 15 patients, LBB potential was recorded in 10 of 15 (66.7%) patients with AVR/TAVR and five of seven (71.4%) patients without AVR/TAVR. No LBB potential was found in four patients with LBBB and three patients with temporary PM dependency due to complete block without escape rhythm. A typical ECG morphology of right BBB was recorded during the lead implantation for LBBP in 20 patients (Figure 2). In two patients with failed LBBP, the paced QRS duration was 144 ms and 146 ms without RBBB pattern. Possible explanation may be related to the local hypertrophic myocardium. The final paced QRS duration was 124.5 ± 13.8 ms, while the baseline QRS duration was 120.0 ± 32.6 ms (*p* = 0.346). The mean stim-LVAT was 70.6 ± 8.1 ms. LBBP was successfully achieved in four of five patients with LBBB. Successful correction of LBBB and narrowing QRS duration were recorded in these four patients (Figure 3).

### Complications and Follow-Up

No procedure-related complications occurred during the implantation procedure. Pocket hematoma or infection, loss of capture, lead dislodgment, or septal perforation was not observed during the mean follow-up of 16.1 ± 10.8 months (ranged from 3 to 33 months). Pacing threshold, R-wave amplitude, and lead impedance at implant and follow-up were described in Table 2. Lead parameters, including pacing threshold, R-wave amplitude, and lead impedance, were stable during the follow-up period. Of our series, 15 out of 22 patients fulfilled the 12 months of follow-up, and echocardiographic data were noted. No significant differences in LV end-diastolic diameter (47.8 ± 7.5 mm vs. 44.1 ± 4.5 mm, *p* = 0.12) and LVEF (63.3 ± 8.2% vs. 62.6 ± 3.4%, *p* = 0.83) were found compared with those at baseline. The degree of TR noted was detailed as follows: none in three (20%), mild in 10 (66.7%), and moderate in two (13.3%) patients. No significant exacerbation of TR was observed compared to baseline. The obvious restriction of leaflet motion by the LBBP lead was not found in any patient.

**Table 2:** Pacing parameters at implant and follow-up.

**Pacing parameters**						
	**Implant**	**1 month(*n* = 22)**	**3 months (*n* = 22)**	**6 months(*n* = 20)**	**12 months (*n* = 15)**	**24 months(*n* = 8)**
Ventricular pacing burden (%)	-	97.75 ± 9.42	98.42 ± 8.52	98.21 ± 7.53	97.64 ± 8.12	98.65 ± 6.45
Threshold at 0.5 ms (V)	0.60 ± 0.16	0.68 ± 0.44	0.76 ± 0.41	0.71 ± 0.17	0.86 ± 0.36	0.91 ± 0.30
R-wave amplitude (mV)	11.9 ± 5.5	16.5 ± 4.0	15.1 ± 6.8	15.8 ± 3.5	16.2 ± 2.3	14.9 ± 5.5
Impedance (Ω)	749 ± 117	523 ± 104	516 ± 92	477 ± 65	503 ± 68	515 ± 70

*Values are described as mean ± SD*.

## Discussion

In the present study, we investigated the feasibility and safety of treating patients with PV-induced AV conduction disorders by pacing the LBB. The major findings of this study are described as follows. First, permanent LBBP was safe and feasible in most patients with PVs referred for PM implantation. Second, the location of the PVs could act as an anatomic landmark and facilitated the LBBP lead implantation. Moreover, a low and stable pacing threshold was observed during the mean follow-up of 16.1 ± 10.8 months.

AV conduction disturbance is a common adverse event in patients undergoing PV surgery. The incidence of PM implantation among patients receiving surgical or transcatheter PV replacement has been reported to be between 4 and 17.2% (11–13). As documented in our study, AV block at the level of the AV node was commonly observed in patients with MVR/TVR, while patients with AVR/TAVR tended to develop infranodal block, which is consistent with a previous study (14). RV apical pacing is the traditional treatment for patients complicated with AV conduction disturbances after PV surgery. However, long-term RV apical pacing may induce ventricular electrical and mechanical asynchrony and therefore increase the risk for heart failure and mortality rate, especially for these patients with AV conduction diseases after PV surgery who have a need for significant ventricular pacing (15, 16).

HBP in patients with AV conduction diseases has been considered to be a more physiological form of ventricular activation (5, 17). However, HBP could not recruit the bundle branches and narrow the QRS width due to failure of pacing beyond the site of conduction block in some patients with PVs, especially in patients undergoing TAVR surgery. Furthermore, HBP may lead to an increase in pacing threshold and lead revision (18, 19). Sharma et al. (14) reported that permanent HBP was achieved in 93% of patients with PVs. However, the success rate for HBP was as low as 50% in TAVR patients. De Pooter et al. (20) described the feasibility of HBP in patients with conduction diseases following TAVR. Their results suggested that HBP with LBBB correction was only recorded in 69% of patients with TAVR surgery. LBBB correction threshold was 1.9 ± 1.1 V at 1.0 ms. Sen et al. (21) reported that LBBB could be successfully corrected by HBP in a patient with TAVR. However, the capture threshold was as high as 5 V at 1 ms at the follow-up of 1 month after the procedure. Vijayaraman et al. (10) recently investigated the success rate of His–Purkinje conduction system pacing in 65 patients with TAVR. They described that HBP was successful in 63% of patients, and LBBP was successful in 93% of patients. LBBP was associated with higher success rates and lower pacing thresholds compared with HBP. It can be explained by a more significant involvement at the level of a more distal conduction system in TAVR patients (22). In our study, all patients with TAVR developed infranodal disease, which is consistent with the abovementioned studies. Therefore, TAVR patients generally tend to develop infranodal block, and lower success in HBP achievement may be expected in this patient population.

LBBP is a novel pacing modality aimed at pacing the conduction system beyond the site of conduction block in most patients with His–Purkinje disease (23). Several studies have reported that LBBP can offer a favorable ventricular mechanical synchrony by the rapid recruitment of left His–Purkinje system, which is similar to HBP (24, 25). Therefore, a more distal recruitment of conduction system in LBBP may provide significant advantage in success rate compared with HBP. Guo et al. (26) reported LBBP in 20 patients with PVs. In their study, LBBP was successfully achieved in four patients with TAVR with a short-term follow-up period of 10.4 ± 5.9 months. In our study, among patients undergoing TAVR, the success rate for LBBP was 80% with a low and stable threshold during the follow-up period of 16.1 ± 10.8 months. We failed to deploy the lead tip to the side of the septum in one patient with TAVR because of local hypertrophic myocardium. Therefore, LBBP may be considered a promising pacing technique especially in patients with TAVR.

HBP in patients with TVR may be challenging because the valve may obstruct the access to His bundle region, which makes a successful HBP difficult. Furthermore, HBP may result in high pacing threshold in some cases (27). Guo et al. (26) also described successful LBBP in four patients with TVR. However, tricuspid valve regurgitation after LBBP procedure was not evaluated in their study. In our study, seven patients with TVR were enrolled for analysis. Successful LBBP was performed in all of them, and no significant worsening of tricuspid valve regurgitation was noted in these patients during the follow-up visit. Therefore, LBBP is feasible and safe in patients with TVR. However, the PV may still limit the ability to steer the sheath, and the significantly enlarged right atrium in some patients is another challenge that needs to be overcome during the procedure. Application of adjustable sheath or prefabricating the sheath during the procedure is a useful way to ensure a successful procedure. Furthermore, the presence of TVR can serve as a radiographic marker and facilitate the location of LBBP. In this study, the average distance from the final site of LBBP was 23.0 ± 3.2 mm distal and septal to the TVR.

LBBP has been considered the physiological pacing modality to date. Hou et al. (24) confirmed the feasibility and favorable cardiac synchrony of LBBP. In their study, LBBP was successfully achieved in 90% of patients. LBB potential was recorded in 61% of patients. Vijayaraman et al. (9) prospectively evaluated the effectiveness of LBBP in bradycardia or heart failure patients. LBBP was successful in 93 of 100 patients, and LBB potential was found in 63 patients. Li et al. (8) also reported a similar success rate in LBBP. In the present study, a majority (90.9%) of patients with PVs successfully received LBBP. LBB potential was observed in 68.2% of patients. During the follow-up period, the pacing threshold was low, with no loss of capture or lead dislodgment observed. Furthermore, no significant differences in echocardiographic parameters between the baseline and follow-up visit were found despite high pacing burden with LBBP. Therefore, LBBP can be applied safely and feasibly in different patient populations, even in patients with PVs.

### Study Limitations

The study was limited by its retrospective single-center design and relatively small sample size. Further randomized studies with a large sample may be needed to confirm our findings. Only 13.6% had coronary disease, and success rates may not be as high in patients with prior septal infracts.

## Conclusion

Permanent LBBP is feasible and safe in patients with PVs. The location of AVR and TVR may serve as landmarks for guiding the final site of the LBBP. A low and stable capture threshold can be obtained during the follow-up visit.

## Data Availability Statement

The raw data supporting the conclusions of this article are available from the corresponding author upon reasonable request.

## Ethics Statement

The studies involving human participants were reviewed and approved by Ethic committee of Guangdong Provincial People's Hospital. The patients/participants provided their written informed consent to participate in this study. Written informed consent was obtained from the individual(s) for the publication of any potentially identifiable images or data included in this article.

## Author Contributions

All authors listed have made a substantial, direct and intellectual contribution to the work, and approved it for publication.

## Conflict of Interest

The authors declare that the research was conducted in the absence of any commercial or financial relationships that could be construed as a potential conflict of interest.

## Publisher's Note

All claims expressed in this article are solely those of the authors and do not necessarily represent those of their affiliated organizations, or those of the publisher, the editors and the reviewers. Any product that may be evaluated in this article, or claim that may be made by its manufacturer, is not guaranteed or endorsed by the publisher.
